# Time Series Visualizations of Mobile Phone-Based Daily Diary Reports of Stress, Physical Activity, and Diet Quality in Mostly Ethnic Minority Mothers: Feasibility Study

**DOI:** 10.2196/11062

**Published:** 2018-11-05

**Authors:** W Scott Comulada, Dallas Swendeman, Roxana Rezai, Nithya Ramanathan

**Affiliations:** 1 Department of Psychiatry and Biobehavioral Sciences University of California, Los Angeles Los Angeles, CA United States; 2 Nexleaf Analytics Los Angeles, CA United States

**Keywords:** changepoint, diet quality, mobile phone, moving average, physical activity, stress, time series

## Abstract

**Background:**

Health behavior patterns reported through daily diary data are important to understand and intervene upon at the individual level in N-of-1 trials and related study designs. There is often interest in relationships between multiple outcomes, such as stress and health behavior. However, analyses often utilize regressions that evaluate aggregate effects across individuals, and standard analyses target single outcomes.

**Objective:**

This paper aims to illustrate how individuals’ daily reports of stress and health behavior (time series) can be explored using visualization tools.

**Methods:**

Secondary analysis was conducted on 6 months of daily diary reports of stress and health behavior (physical activity and diet quality) from mostly ethnic minority mothers who pilot-tested a self-monitoring mobile health app. Time series with minimal missing data from 14 of the 44 mothers were analyzed. Correlations between stress and health behavior within each time series were reported as a preliminary step. Stress and health behavior time series patterns were visualized by plotting moving averages and time points where mean shifts in the data occurred (changepoints).

**Results:**

Median correlation was small and negative for associations of stress with physical activity (*r*=−.14) and diet quality (*r*=−.08). Moving averages and changepoints for stress and health behavior were aligned for some participants but not for others. A third subset of participants exhibited little variation in stress and health behavior reports.

**Conclusions:**

Median correlations in this study corroborate prior findings. In addition, time series visualizations highlighted variations in stress and health behavior across individuals and time points, which are difficult to capture through correlations and regression-based summary measures.

## Introduction

Ecological momentary assessment (EMA) [1-3], daily diary, and weekly assessment, hereafter referred to as *intensive longitudinal assessment* (ILA), is a data collection framework that prompts individuals to self-report behaviors and events *in situ* and often as they occur using paper diaries or electronic data collection devices. ILA offers several benefits over traditional in-person assessment that is often conducted in clinic settings, conducted less frequently, and requires recall over longer periods of time, including reductions in social desirability [4,5] and recall biases [1,6,7]. ILA has been used to examine relationships between psychosocial factors, such as stress, cognition, and positive and negative effects, and health behaviors (HBs) over time, such as physical activity (PA) and diet [8-18]. Stress and HB relationships are of special interest as stress increases the susceptibility to cancer, heart disease, stroke, and other diseases [19-23]. HB confers protective effects against these same diseases [20,24-30]. An understanding of the interplay between stress and HB informs the design of healthy lifestyle interventions. ILA is favored over traditional assessment methods because changes in stress levels and HB occur over shorter periods of time (often over days or weeks) than time periods that are queried through retrospective recall [31]. ILA has gained popularity with the proliferation of mobile phones and advances in mobile phone technology as short message service text messaging and mobile survey apps replace paper diaries and other assessment tools of yesteryears, streamlining data collection and reducing participant burden. A proliferation of mobile phone-based studies across disparate fields of research has resulted, including studies on PA and diet [32-35], drug use [36-38], and HIV [39-41].

Amid advances in ILA data collection methods, analytical strategies to evaluate patterns in resulting data streams have yet to catch up. Random effects (RE) regression models (ie, multilevel and mixed-effects models [42,43]) are recommended [14] and commonly used to analyze data from ILA, or *intensive longitudinal data* (ILD), as in the analysis of EMA data to evaluate stress and PA relationships [31]. Similar to standard regression models, RE models include fixed effects or covariates. For ILA data, covariates are included for time in order to model outcome-level changes over time in the overall sample. In addition to fixed effects, RE models for ILD include RE for time that varies across individuals, and in doing so, allow for individual-level time trends to be estimated. By capturing variations at the individual level, RE models also adjust SE estimates for proper statistical inference. Walls and Schafer [44] adapted RE models for ILD analysis. ILD models provide the ability to analyze within-person effects over time with greater granularity than traditional RE models. Yet, the strength of both traditional RE and ILD models lies in their ability to evaluate between- (eg, sociodemographic) and within-person fixed effects (eg, time trends) that are averaged across individuals, while adjusting for between-person variation through RE.

RE model summaries typically present fixed effect estimates for effects that are averaged across individuals or another level of clustering. For example, studies that treat neighborhoods as clusters use RE models to adjust for neighborhood variation but present neighborhood-averaged effects [45]. When there is interest in health outcome patterns over time at the cluster level (ie, individual level), different analytic approaches are needed; this is especially true for individualized treatment plans that are increasingly utilized for chronic illnesses such as diabetes [46]. N-of-1 trials evaluate individual treatment plans by modifying treatment regimens over the study period based on responses or progress over the same period [47]. Similarly, microrandomized trials randomize treatments and record outcome responses at the individual level over time such as the evaluation of randomly assigned mobile phone health-promoting short message service text messages on PA [48]. Regardless of the individual-level study design, evaluation calls for an analysis of an individual’s data stream over time (hereafter referred to as a *time series*). Moreover, evaluations of both overall and individual-level effects are important for a better understanding of HBs at the population level. For example, PA levels have been shown to be impacted both by Alzheimer’s disease status, as estimated through average effects in older adults [49], and seasonal variations that influence activity at the individual level [50], respectively.

This study fills gaps in the literature and illustrates how time series analyses can be applied to ILD or time series data in the health sciences. Time series analyses have largely been lacking in health sciences and have focused on analyses for single measures when they do occur such as PA accelerometer data from mobile devices [51,52]. We show how time series analyses can be used as an exploratory tool to visualize patterns in single and multiple time series at the individual level. Analyses are illustrated on daily diary data from a pilot study that collected information on stress, PA, and diet quality in mostly ethnic minority mothers [32,53]. Prior studies have explored mental and physical health relationships by applying regression models to ILD [8-18] and data from cross-sectional and longitudinal study designs that collected data over several time points [31,54]. To the best of our knowledge, stress and HB relationships have not been evaluated through time series analyses. In doing so, we highlight insights that time series analyses can provide as a complementary procedure to regression models.

## Methods

### Study Participants

From January 2012 through September 2012, 44 mothers with at least 1 child under the age of 18 years and living at home were recruited to pilot test a mobile app for self-monitoring stress, PA, and diet quality over a 6-month period in Los Angeles, California. Participants were recruited in public venues, such as grocery stores, and through local Web-based parenting groups in the Los Angeles area. The mobile app was designed to help them record stress, PA, and diet quality levels on a daily basis, and in doing so, to self-monitor stress and health-related behaviors. This study was approved by the Institutional Review Board at the University of California, Los Angeles.

### Study Procedures

Once enrolled in the study, participants completed a Web-based baseline assessment to collect sociodemographic characteristics, measures on PA, dietary intake, and perceived stress. Anthropometric measures, including body mass index and blood pressure, and biomarker measures, including C-reactive protein levels and Epstein-Barr virus antibody levels, were also collected. Web-based assessments, anthropometric and biomarker data collection, were repeated at 3 and 6 months after the baseline. Further details on study measures may be found in Comulada et al [32] and Swendeman et al [53].

Following the baseline assessment, participants were assigned Samsung Vibrant smartphones running on the Android operating system version 2.2 or higher. For 6 months, participants received daily time-based prompts on their mobile phones to complete EMA 3 times daily and an end-of-day assessment (daily diary) through a mobile app. Measures encompassed PA, diet quality, and stress in parallel to domains that were captured through Web-based assessment. Participants were also encouraged to use their mobile phone to take pictures of their meals as photographic food records.

Prior analyses examined correlates of adherence to EMA, daily diary reports, and photographic food records [32] and the validity and reliability of measures collected through the mobile app, Web-based assessment, anthropometric measurement, and biomarkers [53]. For this paper, time series analytical methods are illustrated on daily diary measures for stress, PA, and diet quality.

### Sociodemographic and Baseline Characteristics

The average age of 44 study participants was 30.8 (SD 6.4; range 18-43) years. Most participants reported being an ethnic minority; 43% (19/44) reported being of Latina ethnicity and 39% (17/44) reported being nonLatina African American. A third of the participants (14/44, 32%) reported having a high school education or less. Nearly half of the participants (21/44, 48%) reported working part-time or less. On average, participants were obese with a body mass index of 32.1 (SD 7.0) kg/m^2^. Mean systolic (121.6 [SD 14.6] mm Hg) and diastolic (79.3 [SD 9.9] mm Hg) pressures were in normal ranges.

### Daily Diary Measures

#### Stress

Participants were asked, “How stressful was your day overall on a scale of 1-5 (with 5 being very stressful)?” Integer responses from 1-5 were permitted. Five was anchored as “very stressful” in the wording of the question, implying lower values for lower stress levels. Response categories were not explicitly labeled.

#### Physical Activity

Participants were asked, “How many minutes of activity did you do today” regarding the following 3 intensities of PA: light PA (eg, stretching), moderate PA (eg, fast walking), and vigorous PA (eg, running). Total minutes of PA were calculated as the sum of light, moderate, and vigorous PA minutes.

#### Diet Quality

Participants were asked, “How healthy would you rate your eating today, in terms of both quality and quantity, on a scale of 1-5 (with 5 being very healthy)?” Integer responses from 1-5 were permitted. Five was anchored as “very healthy” in the wording of the question, implying lower values for less healthy eating. Response categories were not explicitly labeled.

### Statistical Methods

#### Data Preparation

In preparing data for time series analyses, it is important to acknowledge the assumed data structure. Equal spacing is assumed between adjacent observations over time. If an observation is missing for a specific time point, the time point with the missing observation is dropped from the analysis dataset, and observations on either side of the missing observation are treated as adjacent observations. Therefore, missing data disrupt the assumed data structure. Our *ad hoc* approach to address missing data examines response rates for each participant’s time series and limits analyses to time series with minimal amounts of missing data so that the assumption of equally spaced time points is more tenable.

#### Time Series Analyses

We present 2 time series analyses that are useful for data visualization and exploration. Both analyses were implemented through R [55], a free and downloadable statistical software program. The first analytic method plots smoothed data points for the time series over time instead of the raw data to make it easier to visualize temporal patterns in the data. There are numerous smoothing techniques. See Cowpertwait and Metcalfe [56] for an overview of smoothing techniques implemented in R. We used unweighted and centered *moving averages* as a widely used general-purpose smoothing technique in the absence of *a priori* information on temporal patterns in the time series data. As the name implies, an average of n data points centered on time point t are plotted at t instead of the first data point in the subset of n data points. The interval n is chosen to strike a balance between a large n that smooths out too many variations in the data to provide useful visualizations and a small n that retains too much information from the original data so that patterns are difficult to visualize. We chose n=30, or approximately a month. Moving averages were plotted through the *tseries* package, version 0.10-42 [57].

Visual inspection of moving average plots provides a subjective guide as to points in time where mean levels, such as stress levels, tend to abruptly increase or decrease, that is, where *changepoints* occur. The second analytic method provides a formal statistical algorithm, referred to as *changepoint analysis*, to locate changepoints. Changepoint analysis formulates a maximum likelihood-based test statistic that rejects a null hypothesis that no changepoints are present in the time series data if the statistic is greater than a specified threshold. The maximum likelihood under the alternative hypothesis needs to accommodate the possibility of multiple time points where the changepoint occurs and the possibility of multiple changepoints. Finally, there is no clearly defined manner for choosing a threshold. Different changepoint algorithms have been developed to address these statistical testing complexities. We used the *changepoint* package, version 2.2.2, which contains 3 mainstream algorithms for detecting changepoints—binary segmentation, segment neighborhood, and Pruned Exact Linear Time (PELT) changepoint algorithms. Details are given in the *changepoint* documentation [58]. Briefly, binary segmentation is the oldest and, arguably, the most widely used changepoint algorithm of the 3. Binary segmentation works by first searching for a single changepoint. If a changepoint is found, the time series is bifurcated into 2 segments. The algorithm then checks for changepoints within each segment, and so forth, until no more changepoints are detected. In contrast, the segment neighborhood and PELT algorithms are more precise algorithms that do not condition the detection of additional changepoints on prior changepoints. PELT is the newest algorithm and the preferred algorithm that we used to detect changepoints when possible. We defaulted to binary segmentation if segment neighborhood and PELT algorithms were overly sensitive at detecting changepoints and detected too many changepoints to provide useful visualizations. Changepoint analysis results are presented through plots from the *changepoint* package that superimpose mean levels and changepoints for the time series over the observed values.

## Results

### Analysis Dataset

**Figure 1:**
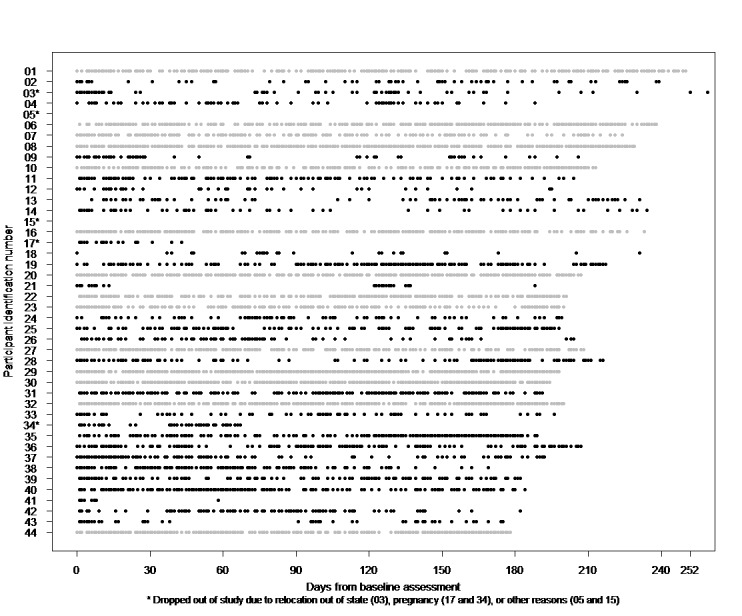
Adherence to filling out mobile phone-based daily diary reports.

Figure 1 shows patterns of compliance to filling out daily diaries for each of the 44 study participants, from the first (01) to the last participant enrolled in the study (44). We retained 32% (14/44) of the time series for analyses from participants who filled out a majority of the possible daily diaries over the follow-up study period based on the visual inspection of Figure 1. Gray and black dots represent time series that were retained and excluded from analyses, respectively. Exclusions included 3 time series for participants who became pregnant or moved out of state during the study period. Time series that were included and excluded in analyses did not significantly differ in terms of participants’ sociodemographic characteristics, anthropometric, and biomarker baseline measures based on chi-square tests for categorical measures (eg, race or ethnicity) and *t* tests for continuous measures (eg, age).

### Correlation Coefficients

As a preliminary analysis, Pearson product-moment correlation coefficients (*r*) were calculated for pairs of concurrent observations for stress and PA, and between stress and diet quality, for each participant. The median association between stress and PA was negative and small in absolute value (*r*=−.14, range: −.39 to.15; n=14 time series). A similarly small and negative median relationship was found between stress and diet quality (*r*=−.08), but exhibited a wider range of correlation coefficient values from −.62 to.65.

### Time Series Analyses

**Figure 2:**
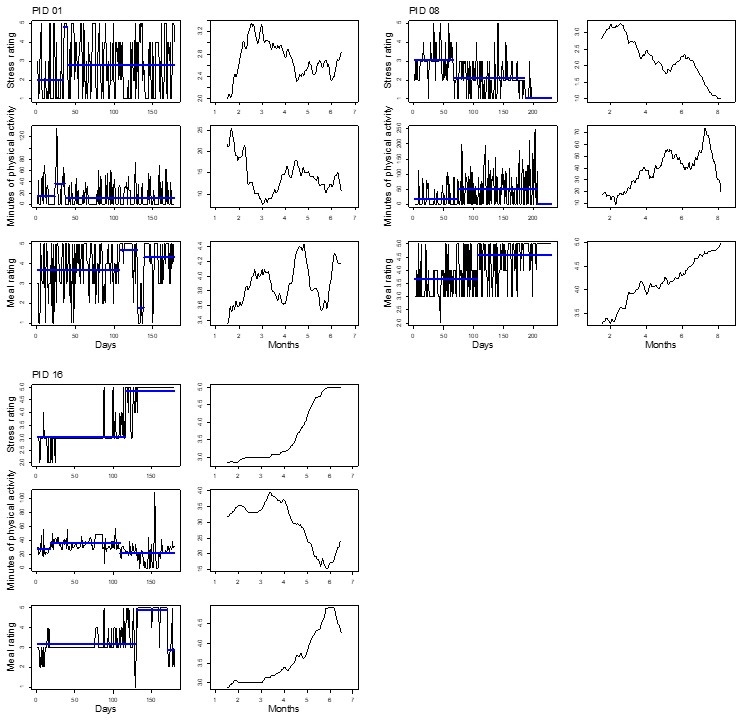
Time series plots for 3 participants exhibiting mean shifts in levels of self-reported daily stress and health behaviors. PID: participant identifier.

**Figure 3:**
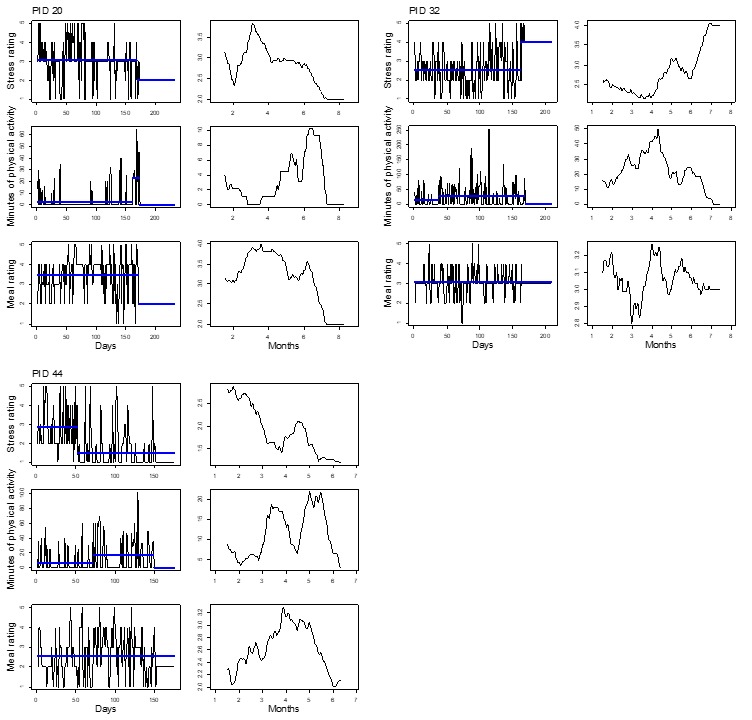
Time series plots for 3 participants exhibiting mean shifts in levels of self-reported daily stress and health behaviors. PID: participant identifier.

**Figure 4:**
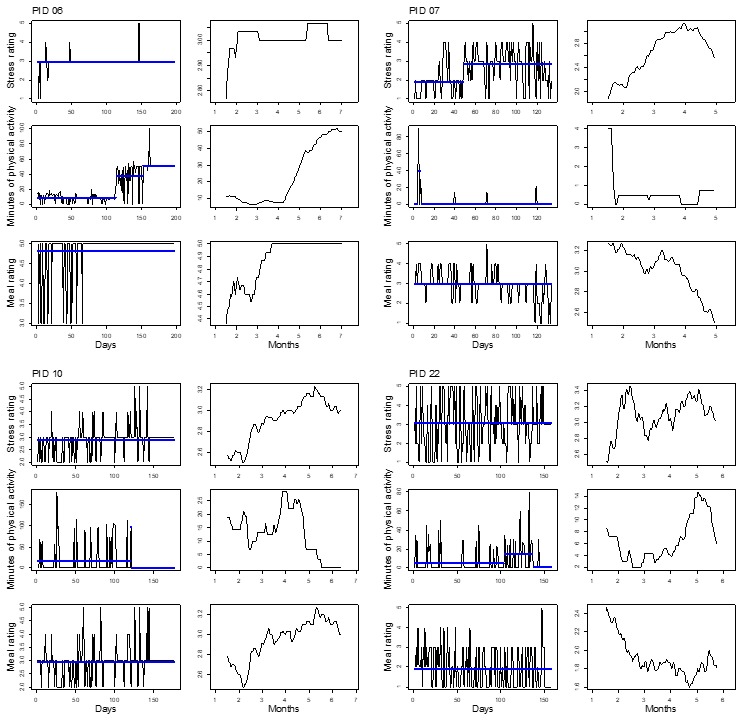
Time series plots for 4 participants not exhibiting mean shifts in levels of self-reported daily stress and health behaviors. PID: participant identifier.

**Figure 5:**
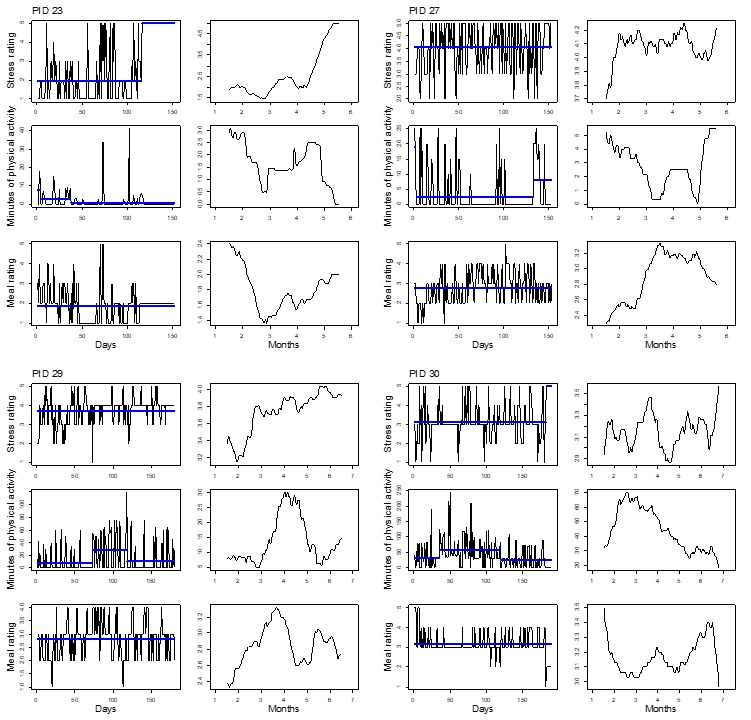
Time series plots for 4 participants not exhibiting mean shifts in levels of self-reported daily stress and health behaviors. PID: participant identifier.

Figures 2-5 show plots for each of the 14 participants retained for analysis. Three pairs of plots are shown for each participant’s time series of stress, PA, and diet quality measurements. For each pair, the plot on the left-hand side shows mean levels indicated by thick horizontal lines and changepoints indicated by line breaks superimposed over the raw time series data. The plot on the right-hand side shows a smoothed line based on moving averages. Changepoint analyses for stress and diet quality were conducted using the PELT algorithm. The PELT algorithm produced too many PA changepoints to be useful for comparison with stress and diet quality changepoints. We resorted to binary segmentation for PA time series and set the maximum number of allowable changepoints to 2; the maximum number of changepoints that were detected for stress in most instances.

Almost all participants exhibited variation in their responses over time, but did not necessarily exhibit abrupt shifts in mean response levels such as reported diet quality for participant 32 in Figure 3; this highlights the utility of different time series visualizations. Moving averages provide for visual evaluation of long-term mean shifts versus abrupt mean level shifts that are detected through the changepoint analysis. To highlight the utility of the changepoint analysis, as well as moving averages, we divided participants between Figures 2 and 3 and Figures 4 and 5 such that Figures 2 and 3 represent participants who exhibited changepoints in both stress and HB (PA or diet quality) levels over the study period (n=6 participants). Participants who did not exhibit changepoints for stress and HB are represented in Figures 4 and 5 (n=8 participants). The division of participants between Figures 2 and 3 and Figures 4 and 5 is another reminder of the potential difficulty for evaluating stress and HB relationships in aggregate when there is heterogeneity in stress and HB relationships across the sample. The remaining discussion focuses on Figures 2 and 3.

Figures 2 and 3 show fairly consistent visual patterns in terms of moving averages for stress and PA levels. Stress and PA levels tend to be inversely related over time. For example, moving average plots for participant 08 show steady declines in stress levels over the study period, matched by a steady increase in PA. Stress and diet quality mean levels are less consistently related, exhibiting inverse relationships, for example, for participant 08, and positive relationships, for example, for participant 16. Changepoint analysis results show that stress and PA changepoints tended to occur at similar points in time; some of the diet quality changepoints did as well. Referring to participant 08 again, we see 3 mean segments for stress, indicating decreasing levels of stress. The first stress changepoint aligns with changepoints for PA and diet quality, indicating increasing levels for both HBs in line with accompanying moving averages. PA also exhibits a third changepoint that indicates a decreasing mean level of PA toward the end of the study.

## Discussion

This paper gave an overview of time series analyses that can be used to better understand individual-level and frequently assessed longitudinal patterns of stress, PA, and diet quality. We highlighted an exploratory approach through visualizations and qualitative descriptions of time series patterns. Analyses began with the calculation of correlation coefficients for each time series as a commonly used statistic to summarize associations. Correlations between stress and PA time series indicated a negative association for 11 of 14 participants in line with negative associations reported in prior studies [31]. Magnitudes of the correlation coefficients were also in line with those reported in prior studies that found negative associations no higher than −.28 to −.42 [31]. The smallest correlation between stress and PA in our study was –.39. It is harder to compare correlations we found between stress and diet quality to prior studies as dietary intake, and not diet quality, is typically assessed. However, a commonality existed in the wide range of correlation coefficients that were found for individual-level associations between stress and PA (−.39 to.15), and between stress and diet quality (−.62 to.65), which underscore the importance of examining stress and HB relationships at the individual level. Stress and HB relational differences across individuals may mask stress and HB relationships that are estimated as average effects across individuals.

Time series visualizations further emphasized variations in stress and HB relationships over time. On a more macro level, time series data yielded 2 groups of individuals based on abrupt shifts in mean levels of stress and HB or a lack thereof in Figures 2 and 5, respectively. Even among participants in Figures 2 and 3 who exhibited changepoints for stress and HB, variations occurred in stress and HB relationships. Stress and PA levels tended to be inversely related over time. Stress and diet quality relationships were more varied, as indicated by moving averages for both measures that sometimes tracked together, in opposite directions, or not at all; diet quality time series did not yield any changepoints for some participants.

It is important to emphasize the exploratory nature of the study; study findings on stress and HB relationships are not confirmatory. A major point of the study findings is that variations in stress and HB relationships indicate the importance of understanding relationships at the individual level. It is also interesting to note that changepoint analyses in Figures 2 and 3 consistently produced 2-3 changepoints for both stress and PA time series that occurred at similar time points. Diet quality changepoints also occurred at similar time points for 3 participants. Commonly occurring changepoints for stress, PA, and diet quality suggest the same underlying causes for longer-term shifts in stress, PA, and diet quality levels; this has implications for interventions that target stress and HBs. A mixed-methods approach may help in understanding the underlying causes of stress and HB shifts or a lack thereof. Through qualitative interviews, participants can be shown visualizations and asked to recall events that precipitated changes. Not surprisingly, moving average patterns and locations as to where changepoints occurred differed across individuals. No shared event existed across individuals that would cause changepoints to align; this highlights a difficulty in RE models that estimate average time trends across individuals. For example, an interrupted time series analysis [59] is an RE modeling equivalent to changepoint analysis but requires an *a priori* specification of where changepoints occur. An *a priori* specification makes sense for interventions, public health policy changes, or other events where changepoints naturally line up across individuals.

In light of the advantages that time series analyses have over RE models for examining variation at the individual level, it is important not to discount the role of RE models in understanding HBs. Population-level inferences are still important. Time series visualizations are useful explorations in line with the notion of preliminary hypothesis-generating studies that inform the development of confirmatory studies with hypothesis-driven statistical tests [60,61]. For example, this study delineated 2 groups of participants based on the presence of changepoints (and a lack of changepoints) in stress, PA, and diet quality levels over time. Our sample size was small, but in a larger sample, subgroup characteristics that relate to variations in stress and HB may emerge and inform the design of large-scale studies that evaluate subgroup effects on stress and HB relationships at the population level.

The potential benefits of time series analyses are tempered by limitations in the assumed time series data structure and need to be contrasted with RE modeling limitations when designing analysis plans. Numerous data points are needed to visualize and evaluate time series in contrast to traditional longitudinal studies that collect data over several time points. Time series studies require careful consideration of both the number of data points to be collected and the length of time over which data points are collected, such as days versus weeks, depending on the measure of interest. For example, Bergman [62] concluded that several days suffice to measure habitual sedentary behavior, whereas close to 6 months are needed to measure habitual vigorous PA with a reasonable degree of accuracy. More studies are needed to determine sufficient numbers of data points and time intervals for accurate inference across different health measures.

In contrast to sensor data, where observations are naturally collected over equally spaced intervals, as in the collection of PA accelerometer data [51,62], ILA tends to be unequally spaced because of missing data. Time series analyses should be applied to ILA data with caution. Methods to address missing time series data, including imputation and model-based approaches [63-65], are not readily available for standard time series routines in software. For example, the *imputeTS* package for R [66] imputes missing observations in time series data but is not integrated with other time series packages in R. Time series analyses should not be automatically ruled out in the presence of missing data, as fruitful inferences can still be made as was the case in this study. Our *ad hoc* approach for dealing with missing data was to only analyze time series analyses with a small amount of missing data so that missing observations would not impact the estimation of moving averages and changepoints to a large degree. Our *ad hoc* approach hinged on 2 main assumptions. Missing data in the time series we retained were minimal and spread out throughout the time series so that adjacent observations in the analysis data were all fairly close in proximity to each other, if not adjacent. Of course, missing data may not be evenly spread throughout the time series; nonresponse may increase over time [32,34,67,68]. Moreover, missing data patterns may differ between time series if participants tend to self-report one HB more than others on a daily basis, for example. This is problematic for commonly used models for multivariate time series (eg, see Tsay [69]), many of which assume equally spaced observations within and between time series.

As the second assumption for our *ad hoc* approach, we assumed missing data to be a random subset of observations from the same distribution as the observed data so that moving average estimates and other time series calculations were not biased by the exclusion of missing data. Missing data were assumed to be *missing completely at random* [70,71]; this is a strong assumption and best addressed by minimizing missing data. When considering study designs that incorporate ILA, it is important to consider buy-in in filling out ILA from the target population so that missing data are minimized. For example, higher rates of filling out ILA have been found in patient [36,38,72] versus nonpatient populations [40,73]. In addition to study design considerations, further statistical development is needed to address missing data in time series analyses and determine the sensitivity to be missing completely at random and other missing data assumptions.

Notwithstanding the limitations, time series analyses provide a starting point where prior studies have left off. In their review paper, Stults-Kolehmainen and Sinha [31] noted different findings for stress and PA relationships across studies, including findings of positive and negative associations, and no association. The same paper provided a reasonable rationale for differences with a focus on study design issues, such as differing levels of rigor and sample sizes. Arguably, different study findings are also attributed, in part, to individual-level differences, some of which were accounted for by regression analyses and some which were not. Time series analyses can help fill in gaps in understanding what traditional regression modeling alone cannot do.

## Abbreviations

EMA: ecological momentary assessment
HB: health behavior
ILA: intensive longitudinal assessment
ILD: intensive longitudinal data
PA: physical activity
PELT: Pruned Exact Linear Time
RE: random effects
